# New era of emerging preoperative chemotherapy in gastrointestinal cancer

**DOI:** 10.1002/ags3.12785

**Published:** 2024-02-28

**Authors:** Keishi Yamashita

**Affiliations:** ^1^ Division of Advanced Surgical Oncology, Research and Development Center for New Medical Frontiers Kitasato University School of Medicine Sagamihara Kanagawa Japan

This issue for the first time includes a JSGS paper, selected in the AGsurg forum held in Hakodate in July 2023. The JSGS papers were selected from four separate sessions on upper GI (gastrointestinal), lower GI, HPB (hepato‐biliary‐pancreatic), and general surgery, from hundreds of applications, and careful, elaborate, and stepwise selection processes comprised many fruitful debates and the consensus of Japanese gastrointestinal surgeons. The initial emerging paper by Narita et al. has reported clinicopathological analysis of 460 cases of carcinoma of the ampulla of Vater (CAV) presented in Hakodate by Dr Hatano from Kyoto University, a corresponding author of this manuscript who was also awarded the AGsurg forum award in the HPB surgery session. The present study consists of a large cohort of patients with CAV as a multi‐institutional study despite its rarity, and the proportion of missing data was extraordinarily small (less than 1% of study population).^1^

They identified six prognostic factors (age, tumor diameter, pathological T factor, portal vein invasion, venous invasion, and pathological N factor) in a multivariate analysis. The prognostic factors identified in this paper were similar with those of the previous series, but the point of remarkable difference was that histological subtype was not an independent prognostic factor. The two large retrospective multicenter cohort studies evaluated the impact of histological subtypes on prognosis in patients with CAV^2^, ^3^; however, 34% and 38% of the study subjects in each study had missing data regarding histological subtype, which could potentially affect the reliability of results. In the present JSGS paper, histological subtype (pancreatobiliary and mixed type) was one of the prognostic factors associated with shorter survival but was not an independent prognostic factor through multivariate analysis. Notably, the current study had only three patients missing data regarding histological subtypes of CAV. Importance of missing data is considered to be claimed in such delicate discussion.

In this research, therapeutic strategy for CAV was also focused on, because standard therapeutic strategy for aggressive CAV has not been established yet. The current analysis was performed out of 80 patients who received postoperative adjuvant chemotherapy (AC), where 63 patients were assigned for propensity score matching (PSM). The results showed no obvious benefit of AC on recurrence free survival, which indicated preoperative chemotherapy is the only remaining potential treatment to improve patient survival of aggressive CAV at present.

Based on such interpretation of the PSM outcomes, the authors thereafter explored preoperative factors potentially predicting the independent prognostic factors (pT ≥ 2, V+, and/or N+) identified in this study, and they were associated with one of the followings: (1) CA19‐9 > 37 IU/mL, (2) ulcerative or mixed‐type appearance, and (3) except for well‐differentiated tumor, or (4) except for intestinal subtype of histology. Intriguingly, preoperative factors such as CA19‐9 and gross appearance were identified, proposing that they can help enrich the potential high‐risk candidate patients for preoperative chemotherapy in the near future. This finding can give not only the member of the JSGS but also world surgeons excellent reference for future research outline in CAV.

The JSGS paper included discussion between authors and discussants, the specialists of each session of the JSGS committee. Professor Kaido and Sho pointed out that AC regimen, including dose intensity and duration, are heterogeneous (S1 alone was the most frequently administered in about 60%), and that it may be important to examine the efficacy according to each regimen of chemotherapy. However, due to rarity of the disease, this critical issue would not be resolved so easily. Professor Sho also commented on the technical interpretation of similar results of relapse‐free survival with overall survival, suggesting AC may have no impact on recurrence. At least, definite evidence that postoperative AC reduces recurrence in CAV has not been obtained in the present study differently from gastric cancer. As the preoperative approach for chemotherapy has recently gained oncological success in aggressive gastrointestinal cancers such as esophageal cancer^4^ and gastric cancer,^5^ consensus of prognostic factors and preoperative factors to predict survival may be important in the future plan for new strategies for aggressive CAV.

This issue includes three original papers describing preoperative chemotherapy in gastric cancer (Morito et al.), and colorectal cancer (Miyashita et al. and Nakagawa et al.). These papers did not describe obvious success of preoperative chemotherapy to improve survival in gastrointestinal cancer.

Morito et al. have described clinical impact of very early recurrence (VER) after preoperative chemotherapy and conversion surgery for stage IV gastric cancer. In this paper, significantly more patients had liver metastasis before initial treatment in the VER group than in the reference group, and VER patients showed the most dismal prognosis, as expected. These findings suggested that after conversion surgery of gastric cancer the VER group may exhibit similar characteristics to liver metastasis. Such clinical uniqueness may in turn be a surrogate phenotype to identify the convergent molecular features to explain chemoresistance in gastric cancer. So clinical phenotypes after neoadjuvant chemotherapy might be a promising clue for gastrointestinal surgeons fighting the most aggressive cancer in the new era.

Miyashita et al. have clarified that immune checkpoint status and oncogenic mutation profiling of rectal cancer after neoadjuvant chemotherapy (KSCC1301‐A2). The effect of neoadjuvant chemotherapy has been associated with increased expression of immune checkpoint molecules, such as PD‐1, and tumor infiltrating lymphocytes profiles representing high CD8/FOXP3 ratio but has not been associated with genomic alterations in new generation sequence (NGS). Therefore, rectal cancer may be expected to be susceptible to combined immunotherapy with chemotherapy. Hence, preoperative chemotherapy could be utilized even as an excellent research tool to clarify the treatment response of clinical cancer cells.

Finally, Nakagawa et al. have reported effects of neoadjuvant chemotherapy for patients with highly conditional colon cancer (obstructive cases) in multicenter propensity score‐matched analysis (YCOG2101). After PSM, 5‐year overall survival tended to be better in neoadjuvant cases (88.5%) than in reference (78.8%), although the difference was not significant (*p* = 0.09). As cancer cells are a kind of microorganism, therapy should be theoretically started in an invisible step to eradicate, and clinical experience in YCOG2101 would be precious and promising for future validation of potential success of preoperative chemotherapy for aggressive colon cancer.

## CONFLICT OF INTEREST STATEMENT

The author declares no conflicts of interest for this article.

## ETHICS STATEMENT

Approval of the research protocol: N/A.

Informed Consent: N/A.

Registry and the Registration No. of the study/trial: N/A.

Animal Studies: N/A.
